# Diagnostic Accuracy of Magnetic Resonance Imaging in Meniscal Tears

**DOI:** 10.7759/cureus.92155

**Published:** 2025-09-12

**Authors:** James Bottomley, Oday Al-Dadah

**Affiliations:** 1 Trauma and Orthopaedic Surgery, South Tyneside District Hospital, South Shields, GBR; 2 Translational and Clinical Research Institute, Faculty of Medical Sciences, Newcastle University, Newcastle Upon Tyne, GBR

**Keywords:** accuracy, arthroscopy, magnetic resonance imaging, meniscal tear, sensitivity, specificity

## Abstract

Background

The knee menisci are fibrocartilaginous structures that play a key biomechanical role in load transmission, shock absorption, stability, and, ultimately, preservation of articular cartilage. Magnetic resonance imaging (MRI) is vital in the assessment of meniscal pathology and guiding subsequent management options. This study aims to evaluate the diagnostic accuracy of MRI for isolated meniscal tears compared to subsequent arthroscopic findings.

Methodology

This observational study screened 334 patients who underwent arthroscopic knee surgery. Preoperative knee MRI scan results using standardized and uniform protocols were compared to arthroscopic surgical findings to assess the sensitivity, specificity, positive predictive value (PPV), negative predictive value (NPV), and accuracy of initial radiological reports.

Results

A total of 79 patients who underwent arthroscopy for isolated meniscal tears were included in the analysis. MRI demonstrated a sensitivity of 90%, specificity of 83%, PPV of 96%, NPV of 63%, and accuracy of 89% for medial meniscal tears. In lateral meniscal tears, preoperative MRI demonstrated a sensitivity of 65%, specificity of 88%, PPV of 68%, NPV of 87%, and accuracy of 81%.

Conclusions

This study demonstrated high diagnostic accuracy of MRI scans for both medial and lateral meniscal tears. However, radiological findings should not be used in isolation, but instead incorporated as a diagnostic adjunct in the wider clinical picture when making therapeutic decisions. Clinical acumen remains the priority in the context of evaluating these knee injuries.

## Introduction

The menisci are fibrocartilage structures located centrally within the medial and lateral compartments of the knee joint between their respective femoral condyle and tibial plateaus [1,2]. Meniscal tears are very common injuries, primarily due to twisting mechanisms, producing a vast array of symptoms, including pain, locking, clicking, catching, and swelling [1]. Due to their diverse presentations, accurate clinical diagnosis can be challenging, with specialized tests including McMurray’s test, joint-line tenderness, and Thessaly’s test being commonly used to aid diagnosis. McMurray’s test, involving slow extension while rotating the knee in flexion, demonstrates a sensitivity of 61% and relatively higher specificity of 84% [3]. Thessaly’s test, performed through twisting of the body while weight bearing on the isolated leg with the knee flexed, has a similar specificity of 87% but a higher sensitivity of 75% when compared to McMurray’s test [3]. Joint-line tenderness positivity has been shown to have equal sensitivity and specificity of 83% [3]. Their lack of sensitivity and sensitivity has led to multiple reviews advising that they should not be used in isolation to diagnose meniscal pathology [3-6].

Magnetic resonance imaging (MRI) is currently the gold-standard investigation for suspected meniscus tears and is frequently used as an adjunct to clinical assessment to aid the diagnosis, with tears appearing as an increased linear signal within the meniscus and extending to a free-edge on at least two images, displaced meniscal flap, meniscal extrusion, altered meniscal shape, and parameniscal cyst [7]. A meta-analysis conducted by Phelan et al. [8] determined that MRI was accurate for diagnosing both medial (89% sensitivity, 88% specificity) and lateral (78% sensitivity, 95% specificity) meniscal tears. However, Anderson [9] identified numerous MRI artefacts and imaging pitfalls, which may suggest the presence of a meniscal tear but cause inaccurate diagnoses.

Furthermore, 10% of patients over 40 years of age with no symptoms affecting their knees demonstrate evidence of a meniscal tear on MRI, rising to 45% in those aged 70-79 years [10]. This has confounded the clinical significance in patients presenting with non-specific knee symptoms, but also accompanying radiological signs of a meniscal tear. Unnecessary surgical treatment of asymptomatic lesions must be avoided due to the risks of surgery and acceleration of secondary osteoarthritis [11]. Therefore, the high accuracy of MRI to determine tear type, severity, and presence of other pathology is important to aid clinicians and patients through a shared decision-making process.

This study aims to evaluate the diagnostic accuracy of MRI scans for isolated meniscal tears compared to subsequent arthroscopic confirmation. MRI scans are hypothesized to have a high sensitivity, specificity, and accuracy for the diagnosis of isolated meniscal tears.

## Materials and methods

This retrospective observational study evaluated the existing clinical practice of the senior author, a consultant orthopedic surgeon specializing in knee surgery. The study was registered with the hospital’s Clinical Effectiveness Department (registration number: CA9828). This research study constituted a part of the first author’s master’s dissertation.

The patients included in this study underwent arthroscopic knee surgery after clinical and radiological evaluation through a specialist knee clinic. Patients were identified through the use of surgical logbooks. All arthroscopic knee surgeries performed between 2013 and 2021 were reviewed using the hospital’s electronic patient record system (MediTech version 6, Medical Information Technology Inc., Westwood, MA, USA). Patient notes were then examined to ascertain what operation had been performed. Operative reports were also reviewed to record various surgical factors, including the presence of a meniscal tear, laterality of the affected meniscus, tear type, and meniscal tear location. Any patients with concurrent anterior cruciate ligament, posterior cruciate ligament, or lateral collateral ligament tears were excluded to allow for isolated meniscal pathology to be investigated.

MRI scans were obtained using a 1.5 Tesla GE Healthcare SIGNA Artist MRI scanner (GE HealthCare, Chicago, IL, USA). The MRI sequences were obtained as per standard knee protocols used by the radiology department, which included sagittal, coronal, and axial proton density fat-saturated sequences alongside a sagittal T1-weighted sequence. All MRI scans were reviewed and reported by a consultant radiologist to ensure high validity of conclusions. MRI findings were then cross-referenced with the intraoperative results to assess the degree of agreement between the two modalities. MRI scans were analyzed using the Picture Archiving and Communication System (PACS) (Centricity version 6, GE Healthcare, Chicago, IL, USA).

Statistical analysis

Statistical analysis was performed using confusion matrices. Separate matrices were created for medial and lateral meniscal tears, with calculation of the MRI’s sensitivity, specificity, positive predictive value (PPV), negative predictive value (NPV), and accuracy to each corresponding laterality. A combined confusion matrix was also illustrated, allowing further analysis into the presence of combined bilateral tears involving both the medial and lateral menisci. Statistical analysis was performed using SPSS for Windows version 26.0 (IBM Corp., Armonk, NY, USA).

## Results

A total of 334 patients were screened using the inclusion and exclusion criteria, following which 79 patients were included in the final analysis. Baseline demographics of the study cohort are summarized in Table 1.

**Table 1:** Patient demographics. SD: standard deviation

Demographic		Number	Percentage (%)
Number of patients		79	-
Age (years) (mean)		58	SD = ±14
Gender	Female	32	41
	Male	47	59
Knee laterality	Left	39	49
	Right	40	51
Smoking	Yes	5	6
	No	74	94
Meniscal tear location	Medial	57	72
	Lateral	14	18
	Both	8	10

**Figure 1:**
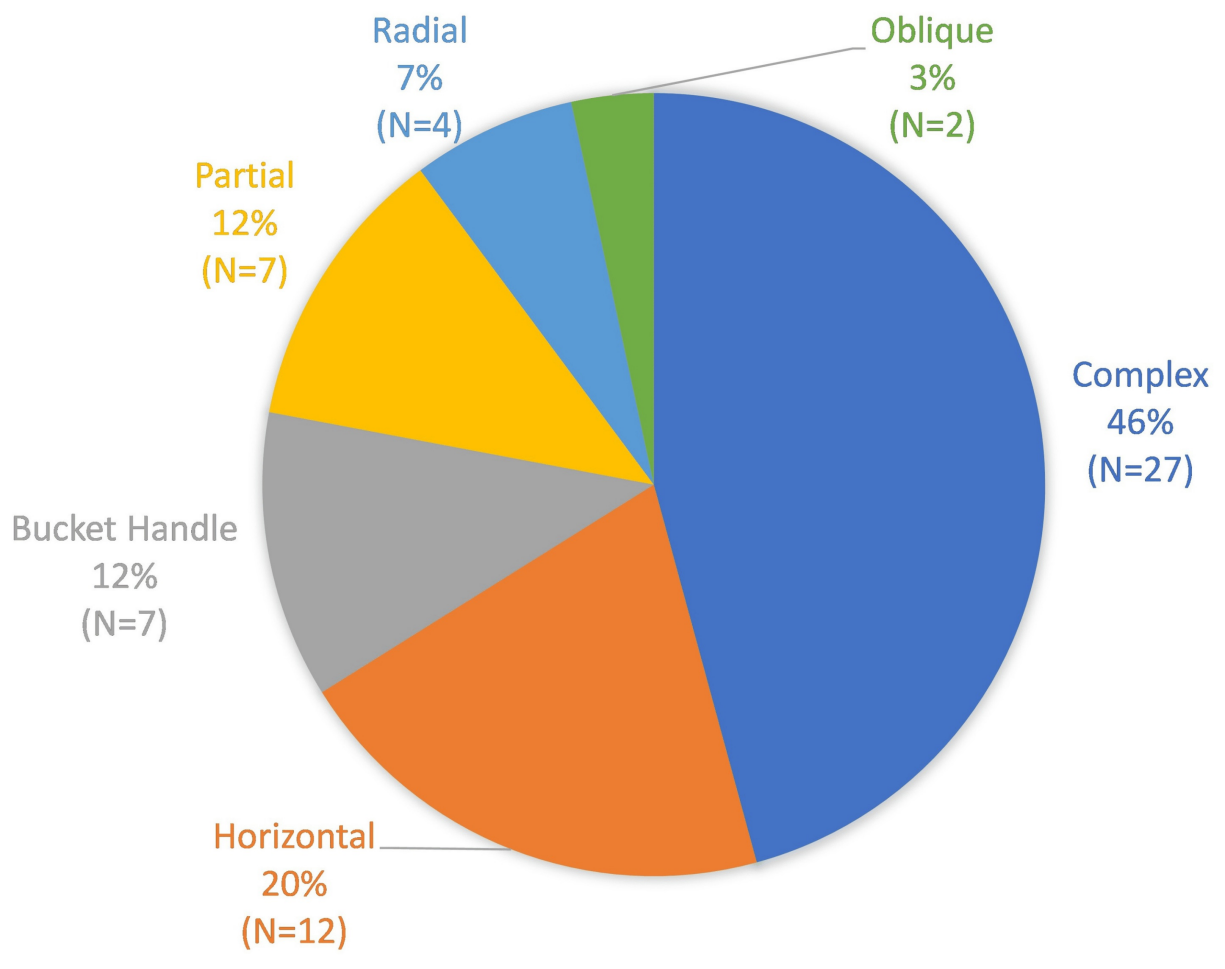
Meniscal tear patterns. Total number of patients = 59.

Figure 1 demonstrates the variety of meniscal tear subtypes. Almost half (n = 27, 46%) of the patients were found to have a complex tear pattern, followed by horizontal tears (n = 12, 20%), with an equal proportion of patients demonstrating bucket handle and partial meniscal tears (n = 7, 12% each). The least frequently observed tear subtypes in this study included radial (n = 4, 7%) and oblique (n = 2, 3%) tears. The specific meniscal tear pattern was not documented in 20 operative notes, resulting in a reduced total of 59 patients having a documented meniscal tear pattern.

Table 2 shows the confusion matrix plot comparing the results of both medial and lateral meniscal tears reported from MRI and arthroscopy. Only 5% (4/79) of patients had complete disagreement between modalities, all when the preoperative MRI indicated no tear was present, but a tear was later found during arthroscopy.

**Table 2:** Confusion matrix: MRI vs. arthroscopy – both medial and lateral meniscal tears. MRI: magnetic resonance imaging

	Arthroscopy					
MRI	N = 79	Medial	Both	Lateral	Neither	Unknown (no notes)
	Medial	44	4	1	0	1
	Both	4	2	1	0	0
	Lateral	2	2	8	0	0
	Neither	2	0	2	0	0
	Unknown (no MRI)	6	0	0	0	0

Table 3 and Table 4 illustrate the diagnostic accuracy of MRI for medial meniscal tears. A high sensitivity of 90% was observed with a slightly lower specificity of 83%. This suggests MRI is a good rule-out test, in which negative results are accurate for medial meniscal tears, but perhaps less accurate as a rule-in test. MRI also demonstrated a high PPV of 96% for medial meniscal tears, but a lower NPV of 63%. By only assessing operative patients, it can be expected that this study would report a greater PPV and smaller NPV than one including a wider population (i.e., normal knees). A high accuracy level of 89% was observed for medial meniscal tears.

**Table 3:** Confusion matrix: MRI vs. arthroscopy – medial meniscal tears. MRI: magnetic resonance imaging

	Arthroscopy			
MRI	N = 79	Tear	No tear	Unknown (no notes)
	Tear	54	2	1
	No tear	6	10	0
	Unknown (no MRI)	6	0	0

**Table 4:** MRI vs. arthroscopy – medial meniscal tears: statistical analysis. MRI: magnetic resonance imaging; PPV: positive predictive value; NPV: negative predictive value

Sensitivity	Specificity	PPV	NPV	Accuracy
90%	83%	96%	63%	89%

The confusion matrix shown in Table 5 and the data in Table 6 illustrate the diagnostic precision of MRI for lateral meniscal tears. Contrary to that of the medial meniscus, a lower sensitivity of 65% was observed with a greater specificity of 88%. This suggests MRI to be a good rule-in test for lateral tears, where positive results are accurate, but less beneficial as a rule-out test. In contrast to medial tears, MRI in lateral meniscal tears showed a greater NPV of 87%, but a lower PPV of 68%. The difference in predictive values is partially attributable to a greater proportion of medial meniscal tears included in the study. The accuracy of MRI scans for lateral meniscal tears was shown to be 81%.

**Table 5:** Confusion matrix: MRI vs. arthroscopy – lateral meniscal tears. MRI: magnetic resonance imaging

	Arthroscopy			
MRI	N = 79	Tear	No tear	Unknown (no notes)
	Tear	13	6	0
	No tear	7	46	1
	Unknown (no MRI)	6	0	0

**Table 6:** MRI vs. arthroscopy – lateral meniscal tears: statistical analysis. MRI: magnetic resonance imaging; PPV: positive predictive value; NPV: negative predictive value

Sensitivity	Specificity	PPV	NPV	Accuracy
65%	88%	68%	87%	81%

## Discussion

This study found high sensitivity (90%) and specificity (83%) of MRI in diagnosing medial meniscal tears. Values were consistent with a systematic review and meta-analysis by Phelan et al. [8], which reported MRI to have an 89% sensitivity and 88% specificity. The results for lateral meniscal tears demonstrated a sensitivity 65% and specificity of 88% in this study compared to 78% and 95%, respectively, by Phelan et al. [8]. These lower values can partially be attributed to the majority of meniscal tears affecting the medial side. In this study, 58 medial meniscal tears were identified through arthroscopy compared to only 20 lateral meniscal tears.

However, most studies included by Phelan et al. [8] were deemed to have a high or unclear risk of bias, with concerns over the suitability of patient selection. This study only included patients seen in a specialist knee orthopedic clinic, reducing the risk of bias and ensuring appropriate patient selection. Therefore, the consistency of MRI sensitivity and specificity found between the two studies demonstrates the accuracy of the imaging modality as a diagnostic adjunct, but it should not be wholly relied upon to dictate the treatment pathway.

Higher sensitivities and specificities of MRI findings can be seen in medial meniscal tears for multiple reasons, including the anatomical and biomechanical differences between the medial and lateral menisci. The menisci are adhered to the tibia via coronary ligaments. The popliteus tendon interrupts the peripheral attachments of the lateral meniscus, thus making the lateral meniscus more mobile. The medial meniscus is closely adhered to the medial collateral ligament and joint capsule, and therefore, is more fixed and less mobile, facilitating a higher load-bearing role during normal gait [12]. As a result, increased stresses within a less forgiving mechanical environment result in higher rates of medial meniscal tears, which are typically larger and more evident on MRI.

Discrepancies between the accuracy of MRI for medial and lateral meniscal tears can also be attributed to imaging artefacts. Lateral meniscal tears, particularly in the posterior horn, can potentially be obscured by adjacent structures in the knee, such as the popliteus tendon and meniscofemoral ligaments [12]. The lateral meniscus, particularly the posterior horn, can also be affected by the magic angle phenomenon, in which tears can be obscured due to the orientation of the lateral meniscus in certain knee positions while being scanned [13,14]. The lateral meniscus is also slightly smaller and thinner, which can make partial tears less apparent [15].

Differences in tear configurations between the menisci can also impact the reliability of MRI findings. Medial meniscal tears are often degenerative and vertical or horizontal in older patients, producing stronger and clearer signal lines on MRI [16,17]. In contrast, lateral meniscal tears are often complex, radial, or flap-type tears, which can appear more subtly as contour irregularities, rather than obvious linear signals [16,17].

There is limited literature regarding the accuracy of meniscal tears based on tear type and meniscal location. Suggestions for further research include evaluating MRI diagnostic accuracy pertaining to different meniscal tear patterns and anatomical locations. This information would allow clinicians to be better informed when making treatment-based decisions depending on MRI findings.

A further meta-analysis conducted by Wang et al. [18] included 17 studies assessing the diagnostic accuracy for meniscal tears. For medial meniscal tears, Wang et al. calculated an average sensitivity of 92% and a slightly lower specificity of 90% [18]. This is in keeping with the results of the present study, with a sensitivity and specificity of 90% and 83% respectively. Further similarities are observed with regard to lateral meniscal tears, demonstrating a greater specificity, 95% by Wang et al. and 88% in the present study, and a lower sensitivity, 80% by Wang et al. and 65% in the present study [18]. However, Wang et al. did not exclude any reviews that included patients with concurrent anterior cruciate ligament injuries. A greater degree of trauma in these patients could explain the higher reported values for MRI sensitivity and specificity for diagnosing meniscal tears. The present study provides a unique perspective on the diagnostic attributes of MRI in diagnosing isolated meniscal tears in stable knees.

Results from the present study showed that MRI has a PPV of 96% and an NPV of 63% for medial meniscal tears. This suggests that a positive result on MRI scan for medial meniscal tears can reliably be observed at the time of arthroscopy, but a negative result cannot be used to reliably exclude a medial meniscal tear.

The converse was observed regarding lateral meniscal tears. The results showed a greater NPV of 87%, indicating a negative result on MRI being more likely in keeping with arthroscopy findings, but a lower PPV of 68%. The differences in these outcomes when compared to medial meniscal tears can be attributed to a greater overall prevalence of isolated meniscal tears occurring in the medial meniscus in patients with stable knee ligaments.

The present study provides a unique insight into the PPV and NPV of MRI when assessing meniscal tears. The differences in predictive values of MRI scan results depending on which meniscus is reported to be affected should be considered when planning further treatment.

Compared to arthroscopic findings, the accuracy of MRI in the present study was 89% for medial meniscal tears and 81% for lateral meniscal tears. Despite high levels of accuracy, these figures are comparatively lower than a study by Riel et al. [19], which demonstrated respective accuracy levels of 95% for medial meniscal and 93% for lateral meniscal pathology. The higher accuracy levels calculated by Riel et al. could be attributed to the difference in patient selection. The present study only included patients with isolated meniscal tears, whereas Riel et al. [19] did not exclude patients with concurrent cruciate ligament tears. The greater degree of trauma to patients with other concurrent injuries could suggest more substantive and sizeable meniscal tears, which are more easily detected on MRI scans.

By only assessing patients with isolated meniscal tears, the present study provides a unique insight into the accuracy of MRI scans in patients who may present with ambiguous or atypical symptomology. This is critical to ensure correct patient selection and avoid unnecessary operative intervention. Overall, 5% of patients included in the present study showed no sign of meniscal tear on preoperative MRI but were found to have an identifiable tear during arthroscopy. This shows that MRI scans should not solely be used in isolation to exclude the presence of a meniscal tear. MRI scans remain important radiological investigations for diagnosing meniscal tears, but suggest that the sole reliance on MRI for accurate diagnosis is not appropriate. History and physical examination should always remain the priority in the clinical evaluation of patients presenting with knee pain, with MRI scans serving as a diagnostic adjunct only.

Potential limitations of this study include its retrospective nature and sample size of 79 patients. This study also only included those who were selected to undergo arthroscopic surgery; therefore, their symptoms and meniscal tears may have been more severe, increasing the likelihood of a positive finding. However, the requirement of arthroscopy to correlate radiological findings necessitates the selection of these individuals, which is therefore important. By only including isolated meniscal tears and excluding concurrent cruciate ligament injuries, this study also provides a unique insight into patients with perhaps more nuanced symptomology. This study provides further evidence encouraging the use of MRI as the gold-standard modality for the investigation of isolated meniscal tears.

## Conclusions

This study provides a unique assessment of the diagnostic accuracy of MRI for isolated meniscal tears. The overall accuracy of medial and lateral meniscal tears was comparable, but their sensitivities, PPV, and NPV differed considerably. MRI scans should not be relied upon in isolation but rather incorporated in the wider clinical picture when making diagnostic and therapeutic decisions. MRI is a valuable diagnostic adjunct alongside comprehensive clinical evaluation of knee injuries.
